# Porous tip radiofrequency ablation catheter reduced heart failure‐related complications and healthcare resource utilization in paroxysmal atrial fibrillation patients

**DOI:** 10.1002/joa3.12830

**Published:** 2023-03-16

**Authors:** Alexandru Costea, Bereket Haile, Adam Barone, Maddie Schenthal, Kathrine Romanowicz, Srinivas Rajsheker, Lee Ming Boo, Tina D. Hunter

**Affiliations:** ^1^ Internal Medicine Department University of Cincinnati Cincinnati Ohio USA; ^2^ College of Medicine University of Cincinnati Cincinnati Ohio USA; ^3^ Heritage College of Osteopathic Medicine Ohio University Athens Ohio USA; ^4^ Department of Biology Bellarmine University Louisville Kentucky USA; ^5^ Clinical Science and External Research, Biosense Webster, Inc. Irvine California USA; ^6^ Real World Evidence, CTI Clinical Trial & Consulting Covington Kentucky USA

**Keywords:** atrial fibrillation, catheter ablation, contact force, heart failure, porous tip

## Abstract

**Introduction:**

A 56‐hole porous tip radiofrequency catheter was developed to provide more uniform cooling with less fluid delivery than a prior 6‐hole irrigated design. This study aimed to evaluate the impact of contact force (CF) ablation with the porous tip on complications (congestive heart failure [CHF] and non‐CHF related), healthcare resource utilization, and procedural efficiency in patients undergoing de novo paroxysmal atrial fibrillation (PAF) ablations in a real‐world setting.

**Methods:**

Consecutive de novo PAF ablations were performed between February 2014 and March 2019 by six operators at a single US academic center. The 6‐hole design was used through December 2016 with the 56‐hole porous tip adopted in October 2016. The outcomes of interest included symptomatic CHF presentation and CHF‐related complications.

**Results:**

Of 174 patients who were included, mean age was 61.1 ± 10.8 years, 67.8% were male, and 25.3% had a history of CHF. Ablation with the porous tip catheter significantly decreased fluid delivery (1177 vs. 1912 mL with the 6‐hole design; *p* < .0001). CHF‐related complications within 7 days, particularly fluid overload, were substantially reduced with the porous tip (15.2% vs. 5.3% of patients; *p =* .0281) and the proportion of patients with symptomatic CHF presentation within 30 days postablation was significantly lower (14.7% vs. 32.5%; *p =* .0058).

**Conclusion:**

The 56‐hole porous tip led to significantly reduced CHF‐related complications and healthcare utilization in PAF patients undergoing CF catheter ablation when compared to the prior 6‐hole design. This reduction likely results from the significant decrease in fluid delivery during the procedure.

## Graphical abstract

The role of left atrial appendage occlusion (LAAO) for atrial fibrillation patients that during oral anticoagulant therapy suffer from ischemic events is still a matter of debate. In the absence of a high bleeding risk, a hybrid approach of LAAO + lifelong anticoagulant therapy could be an effective novel strategy

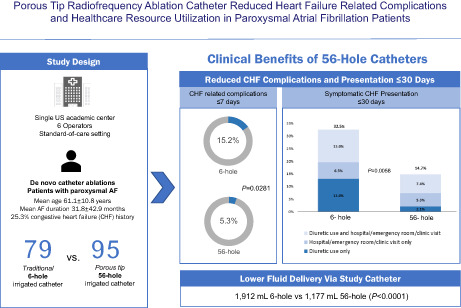

## 1 INTRODUCTION

Radiofrequency (RF) catheter ablation is an effective and minimally invasive treatment option for patients with atrial fibrillation (AF).^1^ The benefits of AF ablation in heart failure (HF) patients with reduced and preserved ejection fractions have recently been demonstrated in the CASTLE‐AF and CABANA trials, respectively.^2^, ^3^ In the former, ablation was associated with a significantly lower occurrence of a composite endpoint of death from any cause or hospitalization for worsening HF compared to medical therapy (28.5% vs. 44.6%, respectively) in patients with a left ventricular ejection fraction ≤35% over a median follow‐up of 37.8 months.^2^ In the latter, after a median follow‐up of 48.5 months, AF ablation was responsible for a 36% relative reduction in a composite endpoint of death, disabling stroke, serious bleeding, or cardiac arrest, as well as a 43% relative reduction in all‐cause mortality compared to drug therapy in a cohort of HF patients who mostly had preserved left ventricular function.^3^ Despite the benefits of AF ablation in HF patients, fluid management during the procedure is critical in these patients to prevent HF‐related complications.

Efforts to improve the outcomes of catheter ablation have led to continuous improvements in catheter design. Compared with nonirrigated catheters, irrigated tips allow for longer and deeper lesions by preserving a low electrode temperature during RF application.^4^, ^5^ Nonetheless, catheter irrigation can result in substantial fluid delivery and risk of fluid overload, particularly in vulnerable patients with preexisting HF.^6^, ^7^, ^8^ A 56‐hole porous tip RF catheter was originally developed to provide more uniform cooling with less fluid delivery than a prior 6‐hole irrigated design in order to reduce fluid overload and associated complications related to congestive heart failure (CHF).

The safety and acute effectiveness of the porous tip design for paroxysmal atrial fibrillation (PAF) ablations have been established for contact force (CF)‐sensing catheters in the SMART‐SF trial.^6^ Compared to the SMART‐AF study of the prior 6‐hole CF catheter,^9^ reductions in procedure time (18.7%), ablation time (14.2%), and total fluoroscopy time (55.2%) were observed. A recent meta‐analysis also reported significantly lower procedural times and irrigation fluid volume for ablations performed with the porous CF catheter versus those performed with the conventionally irrigated CF catheter.^10^ Complication rates were low and similar for both catheters.^10^

The objective of the current study was to evaluate the impact of CF ablation with the porous tip versus the conventionally irrigated tip on complications (CHF and non‐CHF related), healthcare resource utilization, and procedural efficiency among patients undergoing de novo PAF ablations in a real‐world clinical setting.

## 2 METHODS

### 2.1 Study design

This retrospective study analyzed data on de novo PAF ablations performed by six operators at a single US academic center between February 2014 and March 2019. Data were extracted from the patients' electronic medical records into standardized data collection forms.

This study was determined to meet the criteria for exemption from review by the University of Cincinnati Institutional Review Board on May 30, 2019.

### 2.2 Patient population

The study population consisted of consecutive adult patients aged 21 years or older who underwent a de novo PAF catheter ablation at the study site with one of the two study catheters—THERMOCOOL SMARTTOUCH^®^ SF Catheter (STSF, Biosense Webster Inc.), which incorporates the new porous tip, or THERMOCOOL SMARTTOUCH^®^ Catheter (ST, Biosense Webster Inc.), with the prior 6‐hole design. Patients with a history of persistent AF and/or belonging to a vulnerable population were excluded.

### 2.3 Ablation procedure/workflow

RF ablations were performed using the ST between February 2014 and December 2016 or the STSF from October 2016 onwards, both in conjunction with the CARTO VISITAG™ Module (Biosense Webster Inc.).

Pulmonary veins (PVs) were isolated by wide‐area circumferential ablation using the dragging technique. Respiratory gating was utilized. Contact force between 5 and 20 g was sought while maintaining 35 Watts through the left atrium. Pacing of the PVs was performed to verify lack of capture. Entrance block at each PV was then verified by remapping.

### 2.4 Patient follow‐up

Patients were followed up for a year after ablation for safety, symptoms, and treatments. Routine follow‐up visits were scheduled at 7–10 days, 4–6 weeks, and yearly thereafter.

### 2.5 Study outcomes

The primary outcome was symptomatic CHF presentation, defined as a new diuretic prescription, an increase in dosage of a diuretic to alleviate exacerbation, and/or a CHF‐related hospital, emergency room, or clinic visit within 30 days postprocedure. Secondary outcomes included complications (CHF and non‐CHF related) within 7 days and documented fluid overload within 30 days. Procedural efficiency and ablation details were also of interest.

### 2.6 Statistical analysis

All available baseline patient characteristics, procedural details, and safety outcomes were summarized by descriptive statistics and compared across catheter cohorts using chi‐square tests for categorical measures and t‐tests for continuous measures.

Logistic regression modeling was utilized to compare the likelihood of a symptomatic CHF presentation within 30 days postablation between the catheter cohorts, while simultaneously adjusting for baseline CHF and any additional patient characteristics that were associated with the outcome at a significance level of 0.15.

Statistical analysis was performed using SAS software, version 9.4 (SAS Institute Inc.).

## 3 RESULTS

### 3.1 Baseline characteristics

A total of 174 patients who underwent a de novo PAF catheter ablation at the study site between February 2014 and March 2019 were included in the study. Of these, 79 patients (45.4%) underwent ablation with ST and 95 patients (54.6%) with STSF. Patients were followed for an average of 309 ± 134 days postablation.

Patients' mean age was 61.1 ± 10.8 years and 67.8% were male. The mean preablation AF duration was 31.8 ± 42.9 months. Hypertension was the most prevalent comorbidity (78.7%) and 25.3% of the patients had a history of CHF at baseline.

Most patient characteristics were similar across catheter cohorts (Table 1). However, cardiomyopathy and oral anticoagulation utilization were higher at baseline in the ST versus STSF cohorts, while a finding of left atrial enlargement was less prevalent (35.4% vs. 18.9%, 97.5% vs. 84.2%, and 3.8% vs. 12.6%, respectively). Left atrial enlargement was based on a standardized and automated electrocardiogram criteria consisting of a P wave that was >0.1 s duration in leads I and II and that was biphasic with a marked negative deflection in V1.

**TABLE 1:** Patient baseline characteristics.

Characteristic	ST	STSF	*p*‐value
	(*N* = 79)	(*N* = 95)	
Age at procedure (years)	60.5 ± 11.5	61.5 ± 10.1	.5458
Gender (male)	49 (62.0%)	69 (72.6%)	.1360
Time since AF diagnosis (months)	32.3 ± 47.1	31.4 ± 39.5	.9095
Electrocardiogram abnormality, any	15 (19.2%)	28 (29.5%)	.1209
Right bundle branch block	6 (7.6%)	11 (11.6%)	.3782
Left atrial enlargement^a^	3 (3.8%)	12 (12.6%)	.0387
Ejection fraction prior to procedure			.2669
10%–20%	1 (1.3%)	0 (0.0%)	
20%–30%	1 (1.3%)	7 (7.4%)	
30%–40%	5 (6.5%)	4 (4.2%)	
40%–50%	8 (10.4%)	8 (8.4%)	
>50%	62 (80.5%)	76 (80.0%)	
CHA_2_DS_2_‐VASc score	2.9 ± 1.9	2.5 ± 1.5	.0919
Comorbidities			
Congestive heart failure	24 (30.4%)	20 (21.1%)	.1588
Hypertension	61 (77.2%)	76 (80.0%)	.6549
Stroke/TIA	17 (21.5%)	11 (11.6%)	.0756
Vascular disease	23 (29.1%)	24 (25.3%)	.5690
Diabetes	17 (21.5%)	21 (22.1%)	.9257
Cardiomyopathy	28 (35.4%)	18 (18.9%)	.0140
Prior antiarrhythmic drug(s)	34 (43.0%)	54 (56.8%)	.0698
Anticoagulants at time of procedure	77 (97.5%)	80 (84.2%)	.0034

*Note*: Results are displayed as *n* (%) or mean ± standard deviation and include only nonmissing values *p*‐values are based on chi‐square tests for counts and *t*‐tests for means. Abbreviations: AF, atrial fibrillation; ST, THERMOCOOL SMARTTOUCH^®^; STSF, THERMOCOOL SMARTTOUCH^®^ SF; TIA, transient ischemic attack.

^a^ Left atrial enlargement was based on a *P* wave of more than 0.1 s duration in leads I and II that was biphasic with a marked negative deflection in V1 per automated electrocardiogram reading.

### 3.2 Procedural details/efficiency

Fluid delivery via the ablation catheter was significantly reduced with STSF (1177 vs. 1912 mL for ST; *p* < .0001) (Table 2), representing a decrease of 38.4%. These results were consistent for the subset of cases that had no additional ablation performed beyond PVI. No other statistically significant difference between cohorts was observed.

**TABLE 2:** Procedural detail.

Characteristic	ST	STSF	*p*‐value
	(*N* = 79)	(*N* = 95)	
Additional ablation targets beyond PVI	20 (25.3%)	16 (16.8%)	.1695
Roofline	19 (24.1%)	12 (12.6%)	‐
Posterior mitral annular line	5 (6.3%)	3 (3.2%)	‐
Anterior mitral annular line	7 (8.9%)	2 (2.1%)	‐
Box lesions	7 (8.9%)	10 (10.5%)	‐
Foley catheter use	69 (87.3%)	87 (91.6%)	.3608
Fluid delivered via ablation catheter (mL)			
All procedures	1912 ± 799	1177 ± 428	<.0001
PVI‐only (ST: *N* = 59, STSF: *N* = 79)	1889 ± 779	1159 ± 400	<.0001
Length of stay postprocedure (h)	34.2 ± 20.5	33.9 ± 40.9	.9520

*Note*: Results are displayed as *n* (%) or mean ± standard deviation and include only nonmissing values *p*‐values are based on chi‐square tests for counts and *t*‐tests for means. Abbreviations: PVI, pulmonary vein isolation; ST, THERMOCOOL SMARTTOUCH^®^; STSF, THERMOCOOL SMARTTOUCH^®^ SF.

### 3.3 Primary outcome: Symptomatic CHF presentation

**FIGURE 1:**
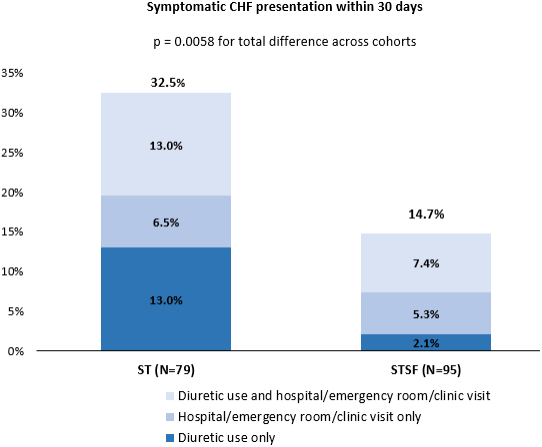
Symptomatic CHF presentation within 30 days.

The primary outcome of symptomatic CHF presentation within 30 days postablation was significantly lower in the STSF cohort than the ST cohort (14.7% vs. 32.5%; *p =* .0058) (Figure 1). This reduction persisted after controlling for baseline CHF and age via multivariable logistic regression (odds ratio = 0.37; *p =* .0103) (Table 3). No additional patient characteristics or procedural details were significantly associated with this outcome.

**TABLE 3:** Multivariable logistic regression model of symptomatic congestive heart failure presentation within 30 days.

Parameter	Effect	Odds ratio	*p*‐value
Ablation catheter	STSF vs. ST	0.37	.0103
Congestive heart failure at baseline	Yes vs. No	2.87	.0091
Age at ablation procedure	Per 10‐year increase	1.45	.0583

Abbreviations: ST, THERMOCOOL SMARTTOUCH^®^; STSF, THERMOCOOL SMARTTOUCH^®^ SF.

### 3.4 Safety/complications

CHF‐related complications within 7 days were significantly reduced after introduction of the porous tip, from 15.2% of patients in the ST cohort to 5.3% in the STSF cohort (*p =* .0281) (Table 4). The greatest contribution to the reduction in CHF‐related complications was a drop of over four‐fold in cases of documented fluid overload within 7 days postprocedure (3.2% STSF cohort vs. 13.9% ST cohort). The incidence of this complication remained significantly lower in the STSF cohort through 30 days of follow‐up (12.6% vs. 26.0% in the ST cohort; *p =* .0254).

The two catheter cohorts were comparable with respect to non‐CHF complications within 7 days (STSF 17.9% vs. ST 16.5%). Groin hematoma and pericardial effusion were the most common non‐CHF complications across both cohorts.

**TABLE 4:** Acute non‐CHF and symptomatic CHF complications.

Complications	ST	STSF	*p*‐value
	(*N* = 79)	(*N* = 95)	
Foley catheter complications	5 (6.3%)	5 (5.3%)	.7636
CHF complications within 7 days	12 (15.2%)	5 (5.3%)	.0281
Fluid overload	11 (13.9%)	3 (3.2%)	‐
Shortness of breath requiring therapy	3 (3.8%)	3 (3.2%)	‐
Documented worsening heart function	3 (3.8%)	2 (2.1%)	‐
Non‐CHF complications within 7 days	13 (16.5%)	17 (17.9%)	.8024
Arteriovenous fistula	0 (0.0%)	1 (1.1%)	‐
Groin hematoma	7 (8.9%)	6 (6.3%)	‐
Stroke	1 (1.3%)	0 (0.0%)	‐
Pericardial effusion	4 (5.1%)	11 (11.6%)	‐
Cardiac tamponade	1 (1.3%)	2 (2.1%)	‐
Documented fluid overload within 30 days	20 (26.0%)	12 (12.6%)	.0254

*Note*: Results are displayed as *n* (%) and include only nonmissing values *p*‐values are based on chi‐square tests. Abbreviations: CHF, congestive heart failure; ST, THERMOCOOL SMARTTOUCH^®^; STSF, THERMOCOOL SMARTTOUCH^®^ SF.

### 3.5 All‐cause and cardiovascular‐related hospitalizations

**FIGURE 2:**
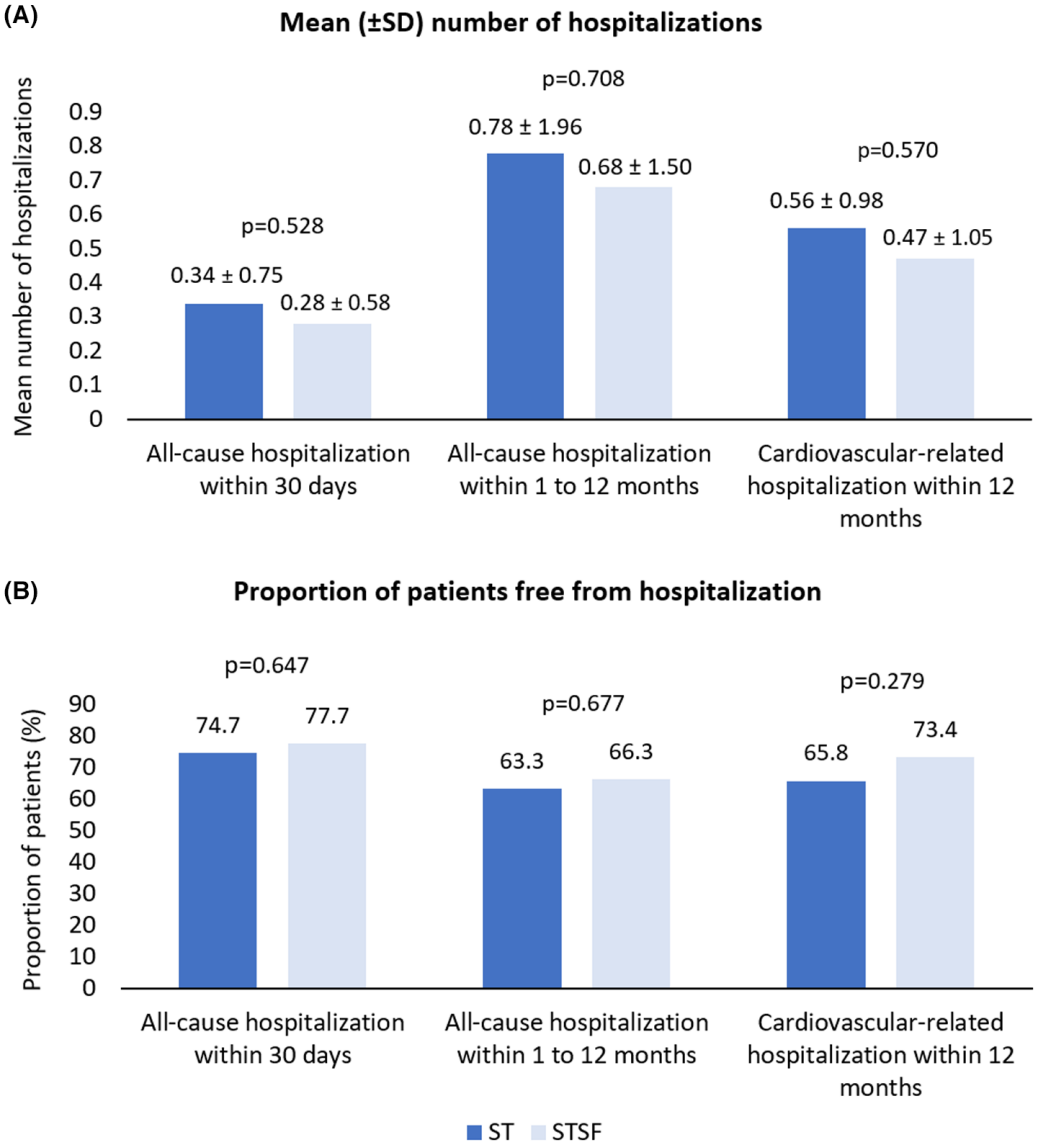
All‐cause and cardiovascular‐related hospitalizations after ablation procedure. SD, standard deviation; ST, THERMOCOOL SMARTTOUCH^®^; STSF, THERMOCOOL SMARTTOUCH^®^ SF.

The majority of patients were free from all‐cause hospitalizations within 30 days (76.4%), all‐cause hospitalizations after 30 days through 12 months (64.9%), and cardiovascular‐related hospitalizations within 12 months postablation (70.1%), with rates similar between STSF and ST cohorts (Figure 2). The mean number of hospitalizations per patient was also similar and low (<1) across all three of these endpoints. No statistically significant differences in freedom from hospitalizations or mean number of hospitalizations were observed across cohorts.

## 4 DISCUSSION

Previous studies have evaluated and compared the safety of ST and STSF ablation catheters.^11^, ^12^, ^13^, ^14^ However, to the best of our knowledge, this was the first real‐world study to evaluate these catheters with respect to acute exacerbations of CHF requiring healthcare utilization and other fluid‐related complications. We found that CHF‐related healthcare utilization was significantly reduced in the STSF cohort, as evidenced by an incidence of symptomatic CHF presentation within 30 days postablation that was less than half of that seen in the ST cohort. This finding aligns with decreases in healthcare resource utilization associated with the porous tip that have been reported by Chinitz et al. for the prior non‐CF‐sensing versions of the porous tip catheters.^1^

In line with published literature,^10^ we observed a significant decrease in fluid delivery via catheter during STSF ablations compared to ST ablations. Reductions ranged from 36.6% to 55.3% in previous studies compared with 38.4% observed in the present study. This reduction was likely responsible for the significant difference in CHF‐related healthcare utilization and complications across catheter cohorts, highlighting the contribution of porous tip catheters to fluid management during AF ablations. Fluid delivery with these catheters (STSF and the newer porous tip QDOT catheter) has continued to decrease to mean values between 382 and 538 mL, which is expected to further reduce CHF‐related complications.^15^, ^16^

The marked decrease in the incidence of fluid overload following STSF ablations—more than 400% within 7 days, and approximately 200% within 30 days—suggests that the porous tip catheter may play an important role in easing the associated impacts on both the patient and payers, particularly considering that a considerable proportion of AF patients have comorbidities that place them at a higher risk of fluid overload.^1^ The potential magnitude of the economic benefit can be estimated from the results of a retrospective study that reported a 29% longer length of stay and 43% higher per‐visit hospital cost among inpatients with diagnosis codes for fluid overload in a large hospital database.^17^

Low and similar complication rates for STSF and ST ablations have been reported by prior studies. Major complications are uncommon with either catheter, with rates varying between 0% and 2.7% in the available literature.^11^, ^12^, ^13^ Rates of minor complications are slightly higher, ranging from 0% to 5%.^11^, ^12^ Consistent with these studies, we found no difference in non‐CHF complication rates across catheter cohorts. The rates observed in the present study (16.5% and 17.9% for the ST and STSF, respectively) were appreciably higher than those previously reported owing to the comprehensive definition used for the data collection. Minor complications (groin hematoma and pericardial effusion), which are rarely reported in other literature,^11^, ^12^ account for the vast majority of recorded complications in our study. As in previous studies,^11^, ^12^, ^13^ major complications (arteriovenous fistula, stroke, and cardiac tamponade) were seldom observed. The higher rate of documented pericardial effusion among patients ablated with the STSF likely stems from the use of intracardiac echocardiography in later years of the study to check for the presence of these events at the end of ablation procedures. Prior to this change in procedure, asymptomatic events would not have been captured. In summary, our results suggest that the use of STSF for the ablation of AF patients, particularly those susceptible to fluid overload, is a safer option when compared to a traditionally irrigated ST catheter.

### 4.1 Limitations

The primary limitations of this study are associated with the nonrandomized retrospective design. ST ablations generally predated those performed with the STSF. Therefore, factors other than the catheter used may have influenced our results. These could have included changes in ablation workflow, increased operator experience over time and/or inclusion of new operators will less experience in the later portion of the study. Confounding by unmeasured variables may have also occurred. Procedure times were not captured in the case records, so could not be summarized for comparison with fluid volumes. Lastly, the single‐site design may limit the generalizability of our findings because of site‐specific contextual factors, namely procedural workflow and levels of operator experience and skill.

## 5 CONCLUSION

In a real‐world setting, a porous catheter tip led to significantly reduced CHF‐related complications and healthcare utilization in PAF patients undergoing CF catheter ablation compared to a traditional 6‐hole irrigated ablation catheter. This reduction likely resulted from the significant decrease in fluid that was delivered via the ablation catheter during the procedure. This finding has the potential to evoke a substantial positive economic impact in addition to the positive impact on patient safety.

## FUNDING INFORMATION

This study was funded by Biosense Webster, Inc.

## CONFLICT OF INTEREST STATEMENT

Alexandru Costea is a speaker for Biosense Webster, Inc. and Biotronik. LM Boo is an employee of Biosense Webster, Inc., the study sponsor. TD Hunter is an employee of CTI Clinical Trial & Consulting, which is a consultant to Biosense Webster, Inc., the study sponsor. The other authors have no conflicts of interest relating to this manuscript.

## ETHICS APPROVAL AND PATIENT CONSENT STATEMENT

This study was determined to meet the criteria for exemption from review by the University of Cincinnati Institutional Review Board on May 30, 2019.

## Data Availability

The data that support the findings of this study are available from the corresponding author upon reasonable request.
